# Wireless control of cellular function by activation of a novel protein responsive to electromagnetic fields

**DOI:** 10.1038/s41598-018-27087-9

**Published:** 2018-06-08

**Authors:** Vijai Krishnan, Sarah A. Park, Samuel S. Shin, Lina Alon, Caitlin M. Tressler, William Stokes, Jineta Banerjee, Mary E. Sorrell, Yuemin Tian, Gene Y. Fridman, Pablo Celnik, Jonathan Pevsner, William B. Guggino, Assaf A. Gilad, Galit Pelled

**Affiliations:** 1 F.M. Kirby Research Center for Functional Brain Imaging, Kennedy Krieger Institute, Baltimore, Maryland 21205 USA; 2 Russell H. Morgan Department of Radiology, Johns Hopkins University School of Medicine, Baltimore, Maryland 21205 USA; 3 Institute for Cell Engineering, Johns Hopkins University School of Medicine, Baltimore, Maryland 21205 USA; 4 Department of Physiology, Johns Hopkins University School of Medicine, Baltimore, Maryland 21205 USA; 5 Department of Otolaryngology, Johns Hopkins University School of Medicine, Baltimore, Maryland 21205 USA; 6 Department of Physical Medicine and Rehabilitation, The Johns Hopkins University School of Medicine, Baltimore, Maryland 21287 USA; 7 Department of Neurology, Kennedy Krieger Institute, Baltimore, Maryland 21205 USA; 8 Department of Biomedical Engineering, Michigan State University, East Lansing, Michigan 48823 USA; 9 The Institute of Quantitative Health Science and Engineering, Michigan State University, East Lansing, Michigan 48823 USA; 10 Department of Radiology, Michigan State University, East Lansing, Michigan 48823 USA

## Abstract

The *Kryptopterus bicirrhis* (glass catfish) is known to respond to electromagnetic fields (EMF). Here we tested its avoidance behavior in response to static and alternating magnetic fields stimulation. Using expression cloning we identified an electromagnetic perceptive gene (*EPG*) from the *K. bicirrhis* encoding a protein that responds to EMF. This EPG gene was cloned and expressed in mammalian cells, neuronal cultures and in rat’s brain. Immunohistochemistry showed that the expression of EPG is confined to the mammalian cell membrane. Calcium imaging in mammalian cells and cultured neurons expressing EPG demonstrated that remote activation by EMF significantly increases intracellular calcium concentrations, indicative of cellular excitability. Moreover, wireless magnetic activation of EPG in rat motor cortex induced motor evoked responses of the contralateral forelimb *in vivo*. Here we report on the development of a new technology for remote, non-invasive modulation of cell function.

## Introduction

The majority of the technologies available to manipulate cellular function in a cell- and spatiotemporal-specific manner demand the use of optics^1–7^, drugs^8–10^, radio-wave heating^11–13^ or ultrasound^14^. However, the quest to identify genes that control cellular function by electromagnetic fields (EMF) that penetrate deep tissue non-invasively is only in its infancy^15,16^. While it is known that various aquatic species use the Earth’s magnetic and electric fields for orientation, navigation and detection of prey and predators^17^, the cellular mechanisms for the same remains unknown. One such organism, *Kryptopterus bicirrhis*, a fresh water fish, contains an ampullary organ dedicated to sense EMF^17^. Evidence suggests that EMF induction results in immediate calcium influx in the electroreceptors cells of the ampullary organs^18^, which are located under the fins. Thus, it is plausible that these electroreceptor cells express proteins that are sensitive to changes in EMF. Fundamentally, identification of a protein that is remotely activated by non-invasive EMF is an unmet need that could complement the growing arsenal of technologies dedicated to the external control of cellular activity *in vivo*.

## Results

### *K. bicirrhis* swim away in response to EMF

**Figure 1:**
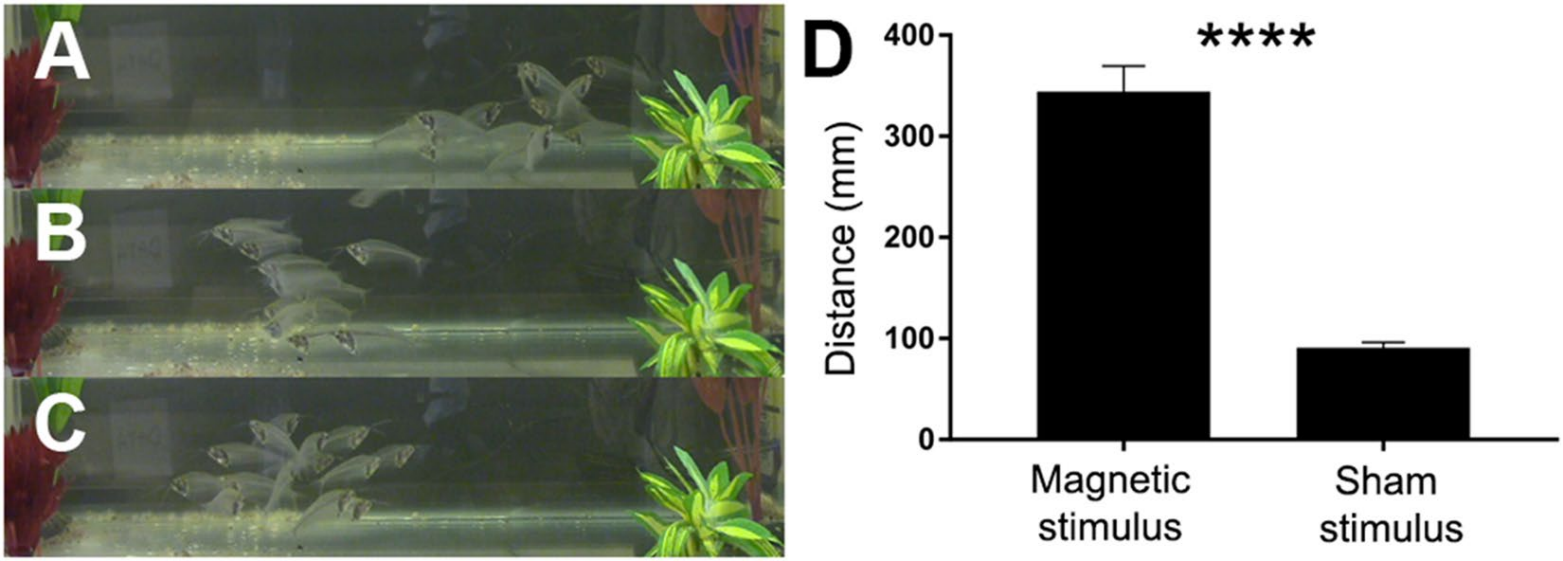
The *K. bicirrhis* swim away in response to EMF. A TMS coil was placed on the right side of the fish tank and induced pulses at a rate of 50 Hz for 5 s. (**A)** Before the stimulation was applied, fish were scattered in the tank. (**B**) During stimulation, all the fish swam away from the stimulation source that was located on the right. (**C**) When stimulation was over the fish swam again in all directions. Fish were rewarded at the end of the trial. (**D**) Static magnetic stimulation induced avoidance behavior (****p < 10^−6^).

We tested the fish behavioral response to static and alternating magnetic fields. Fourteen *K. bicirrhis* were housed in a 30-gallon tank kept at 25 °C (Fig. 1). For behavioral testing, the fish were grouped and placed in a tank. A Neodymium Rare Earth Magnet was placed on one side for 10 s and fish that were 10 mm away were subjected to field strength of 23 mT. Sham stimulus consisted of a plastic object of a similar size. In these trials (n = 10), the fish swam a distance of 344 ± 26 mm in response to the magnetic stimulus which was significantly greater than the distance of 91 ± 5 mm (p < 10^−6^, Student t-test) covered in sham experiments (Fig. 1D). For the alternating magnetic field, a transcranial magnetic stimulation (TMS) system at 250 mT was used. In sham trials the TMS coil was placed on the side of the tank and only an audio-recording of the sound was delivered. During sham trials, the fish were indifferent to the sound produced by the TMS system; upon TMS in experimental trials, all fish swam away from the EMF source. Figure 1A–C (and movies S1 in supplementary data) shows the fish position prior to EMF stimulation and their position in the tank 1 s after the induction of EMF. These results demonstrate that these fish exhibit robust behavioral response induced by static and alternating magnetic stimuli.

### Cloning of the electromagnetic perceptive gene

To identify and characterize the putative EMF-sensitive protein(s), we used expression cloning in *Xenopus laevis (X. laevis)* oocytes^19,20^. We surgically isolated the anal fin containing the electroreceptor organs^21^ from 80 anesthetized *K. bicirrhis* and extracted the total mRNA, from which a cDNA library was constructed. cDNA sub-libraries were screened by two-electrode voltage-clamp (TEVC) in *X. laevis* oocytes^22,23^ for altered current responses to stimulation. One of the sub-libraries exhibited increased, voltage-dependent membrane current in physiological (ND96) and sodium-free (NMDG) solutions. Hence, this sub-library’s 44 cDNA clones were amplified and purified for further screening. The clones were sequenced and all putative genes were compared to the GenBank database. Candidate open reading frames of each putative gene were translated and compared to the protein database. The clones were further divided into smaller sub-libraries and current response was tested by TEVC. This led to the identification of a single open reading frame encoding a protein of 133 amino acids (~15 kDa) that displayed constitutively increased outwardly rectifying current, which we termed electromagnetic perceptive gene (EPG).

Using reverse transcription polymerase chain reaction (RT-PCR) with specific primers we verified that the EPG is indeed constitutively transcribed by the *K. bicirrhis*. (Supplementary Fig. S1). To validate that EPG is translated (i.e. formation of a protein), we produced an affinity-purified polyclonal raised against the putative extracellular domain based on Genscript OptimumAntigen^TM^ Design Program. As can be seen in Supplementary Fig. S1 lanes 1–3, EPG is detected only in tissue extract of the *K. bicirrhis* but not in control tissue from the *Danio Rerio* (Zebrafish) clearly indicating that the EPG is a unique protein to *K. bicirrhis*, that was evolved to perform a distinctive *function*. It is interesting to note, that based on the molecular weight, the EPG appears to be a (homo)dimer.

### Bioinformatic analysis of EPG DNA and protein sequence

A BLASTN^24^ analysis of the EPG cDNA sequence resulted in no matches (unique identity) in the non-redundant genomic DNA database. A TBLASTX analysis of the cDNA sequence, using the database of expressed sequence tags (dbESTs) resulted in statistically significant matches with ESTs from several fishes. From the six possible open reading frames (ORFs) (EMBOSS^25^), the first ORF resulted in a single protein, consistent with the encoded protein sequence from TBLASTX searches. A DELTA-BLAST search of the amino acid sequence produced matches with numerous proteins from the family UPAR_LY6 (accession: *pfam00021*). We constructed a phylogenetic tree of the EPG protein and its homologs (maximum likelihood method, JTT matrix-based model^26^) which showcased the evolutionary relationship of EPG with other proteins in the same superfamily (Supplementary Fig. S2). Multiple sequence alignment using MUSCLE^27,28^ (Supplementary Fig. S3) showed that EPG has a cysteine-rich conserved domain. This domain is homologous to the UPAR_LY6 domain (Supplementary Fig. S3A, amino acid residues bolded in blue).

The predicted secondary structure of EPG includes five beta strands flanked by alpha helices at either terminus (JPred^29^, (Supplementary Fig. S3). We further modeled the membrane orientation of EPG using Polyphobius^30^, which showed a hydrophobic (trans-membrane) N-terminus signal peptide (TMHMM^31^, Signal-Blast^32^) followed by a non-cytoplasmic region (Supplementary Fig. S3). The putative extracellular region includes the five beta strands localized in the UPAR_LY6 domain of the EPG protein. Homology-based modeling of the tertiary structure of the putative extracellular domain of EPG (residues 17–104) predicted a beta sheet enriched disk-like domain with conserved cysteines (SWISS-MODEL^33–36^, cysteines shown as cyan spheres in (Supplementary Fig. S3). An accessibility plot of EPG **(**Supplementary Fig. S3**)** generated using DeepView shows the concave nature of the extracellular domain similar to the UPAR_Ly6 domain of CD59. The concave face of the UPAR_LY6 domain of CD59 is its active extracellular binding domain^37,38^ and bears topological similarity to the Toxin_1 family^39,40^. We hypothesize that intra-molecular disulfide bonds between the conserved cysteines of EPG may help stabilize its non-cytoplasmic domain (similar to those found in CD59). The non-cytoplasmic domain of EPG also contains a predicted N-glycosylation site at residue-77 suggesting that EPG may be a glycosylated protein.

### Cellular and region specific expression of EPG

Human Embryonic Kidney 293T cells (HEK293T) were transfected with pcDNA3.1-EPG. Supplementary Fig. S4 show membrane co-localization between plasma membrane marker pan cadherin (green) and EPG expression (red) in transfected cells vs. un transfected control cells We detected greater co-localization in the EPG transfected cells compared to un-transfected control cells, using both Manders overlap (EPG vs. control: 0.36 vs. 0.32; p-value = 0.024; Supplementary Fig. S4) and Pearson’s correlation^41^ coefficients (EPG vs. control: 0.32 vs. 0.27; p-value = 0.014; Supplementary Fig. S4). Furthermore, stereotaxic injections of pAAV2-CaMKII::EPG-IRES-hrGFP into the lateral ventricle of P1 rats (n = 10) were performed. The subsequent immunostaining in primary somatosensory cortex using an anti-EPG polyclonal antibody (Supplementary Fig. S4**)** showed that EPG can be expressed *in vivo*.

### Mechanistic characterization of the EPG

In order to understand EPG protein’s activity *in vitro* on a mechanistic level, we generated a synthetic gene with the same EPG amino acid sequence but optimized for bacterial expression. We cloned, expressed and purified the EPG on a cobalt column and used it for downstream applications. Circular Dichroism revealed weak alpha helical bands at 208 and 222 nm indicating a small amount of alpha helical structure as supported by the bioinformatics. When the purified protein was exposed to a static magnetic field (25 mT) no conformational changes were observed (Supplementary Fig. S5). This indicates that mechanism of the EPG transduction does not involve significant changes in the structure of the EPG protein. Furthermore, no iron was detected when the purified protein was subjected to digestion with trypsin and chymotrypsin implying that the mechanism of magneto-detection is not dependent on iron-sulfur clusters in the protein.

### EPG characterization in mammalian cells

**Figure 2:**
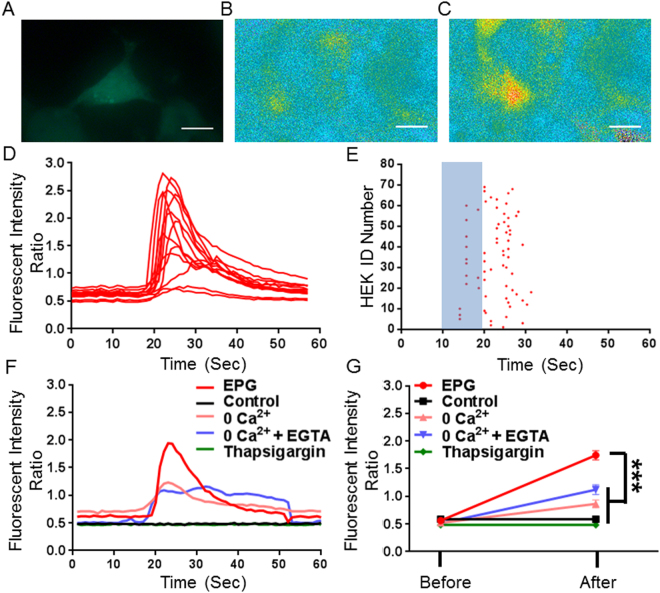
Magnetic stimulation of EPG in HEK293T cells induced significant increases in [Ca^2+]^i. (**A**) HEK293T cells transfected with pLV-CMV-EPG-IRES-hrGFP. (**B**,**C**) *In vitro* calcium images of fura-2/AM loaded cells before (**B**) and after (**C**) 10 s of magnetic stimulation. Black arrow indicates cell body. Scale bar 10 µM. (**D**) Raw data showing the fluorescence increase after magnetic stimulation (blue bar) in cells transfected with EPG. (**E**) Raster plot of transfected cells responding to magnetic stimulation (red dots, n = 68). Cells were classified as responders if the fluorescence signal peak was 10 SD above the mean. (**F**) Data showing kinetics of fluorescence intensity response for individual cells under different conditions. Decreased fluorescence intensity changes were observed in 0 Ca^2+^ and 0 Ca^2+^ + EGTA after magnetic stimulation. No increase in signal was observed for control (non-transfected) and thapsigargin treated cells. (**G**) Data showing the average (±SEM) changes in fluorescence before and after magnetic stimulation for each condition n = 68 (EPG), n = 76 (Control), n = 23 (0 Ca^2+^), n = 9 (0 Ca^2+^ + EGTA) & n = 48 (Thapsigargin).One-way ANOVA, Dunnett’s post-test (F4, 219) = 27.47, P < 0.0001.

HEK293T cells were transfected with a lentivirus construct containing the EPG under CMV promoter (pLV-CMV::EPG-IRES-hrGFP) and calcium imaging using fura-2/AM was obtained two-three days post transfection (Fig. 2A–C). Percent change in fura-2/AM ratio at 340/380 nm excitation was calculated. Cells were subjected to a magnetic field of 50 mT for 10 s induced by a static flat magnet. Out of n = 99 GFP positive cells, 68 cells showed significant increase in [Ca^2+^]i. The mean increase in [Ca^2+^]i fluorescence intensity was 232 ± 20% and occurred 13 ± 0.5 s after stimulus onset (Fig. 2D,E). Data in (Fig. 2D) show variable cellular response in HEK cells from a single transfection in response to magnetic stimulation. Nontransfected cells showed no response to stimulus (n = 76). To determine if the increases in [Ca^2+^]i was due to extracellular origin, EPG-expressing cells were imaged in Ca^2+^-free extracellular imaging solution (n = 23), or in Ca^2+^-free extracellular imaging solution supplemented with Ca^2+^ chelator EGTA (n = 9). Magnetic stimulus resulted in mean [Ca^2+^]i changes of 81 ± 14% and 52.0 ± 3% which were significantly lower compared to normal conditions (*F*_4,219_ = 27.47; p < 0.0001, one-way ANOVA). We then tested if endoplasmic reticulum (ER) calcium release is required in EPG- mediated responses (n = 48). In EPG-expressing cells that were incubated with the inhibitor thapsigargin, magnetic stimulus did not induce any [Ca^2+^]i changes (0.81 ± 1.95%) (Fig. 2F,G). These results suggest that both extracellular and intracellular Ca^2+^ ions are released in response to static magnetic stimulation of EPG.

### EPG characterization in cortical neuronal culture

**Figure 3:**
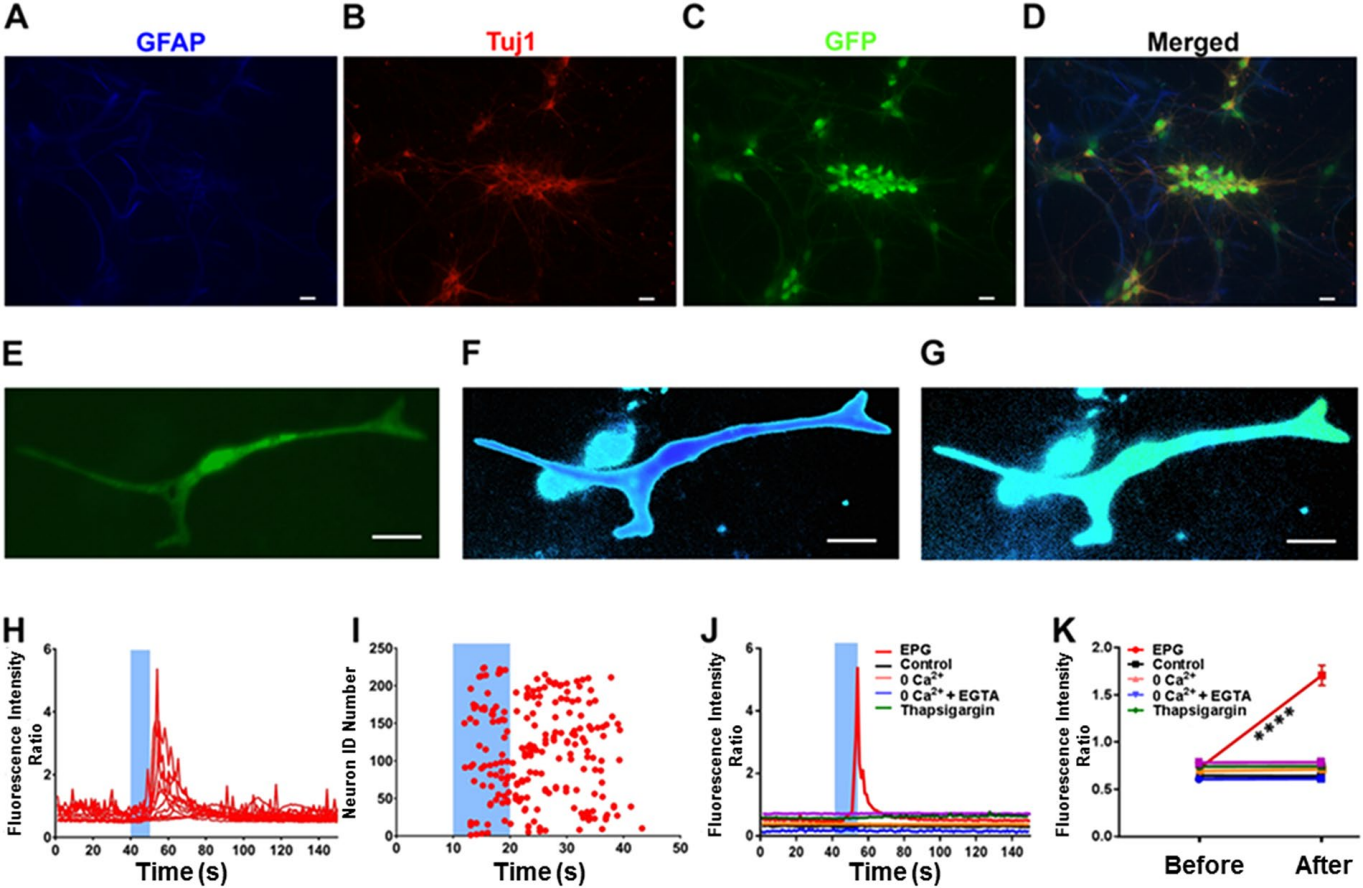
Immunocytochemistry of cortical co-culture and Magnetic stimulation of EPG in neurons. **(A)** The cells are positive for neuronal and glial markers, such as **(A)** GFAP (blue), **(B)** Tuj1 (red) in **(C)** EPG positive neurons (green). **(D)** Colocalization of EPG positive neurons with both GFAP and Tuj1. Primary cortical mixed neuron and glia cultures were transduced with viral constructs for EPG tagged with GFP under the CMV promoter (pLV-CMV::EPG-IRES-hrGFP). (**E**–**K**). **(E)** Primary cortical mixed neuron and glia cultures were transduced with viral constructs for EPG tagged with GFP under the CMV promoter (pLV-CMV::EPG-IRES-hrGFP). Transduced neurons were loaded with fura-2 calcium indicator before **(F)** and after **(G)** 10 s magnetic stimulation of EPG in neurons induces significant increases in [Ca^2+^]i as depicted by a change in the intensity profile. Alternating static magnetic field was applied for 10 s (blue bar). **(H)** Raw data showing the EPG transfected neurons with fluorescence increase after magnetic stimulation (blue bar). **(I)** Raster plot of transfected cells responding to magnetic stimulation (red dots, n = 225). **(J)** Data showing kinetics of fluorescence intensity response for individual cells under different conditions. Increased fluorescence intensity changes were observed in EPG transfected neurons post magnetic stimulation. Different conditions such as O Ca^2+^ (tyrodes solution with Ca^2+^ removed), O Ca^2+^  + EGTA, thapsigargin, mCherry & non-transfected cells show no increase in signal after magnetic stimulation. **(K)** Data showing the average (±SEM) changes in fluorescence before and after magnetic stimulation for each condition n = 225 (EPG), n = 495 (Control), n = 475 (O Ca^2+^), n = 175 (O Ca^2+^ + EGTA), n = 308 (Thapsigargin) & n = 80 (mCherry). Significant increases in [Ca^2+^]i compared to baseline values were measured in neurons expressing EPG-GFP (*p < 0.0005, Student’s *t* test).

Cortical rat co-culture neurons were transduced with a lentiviral construct containing the EPG under the CMV promoter (pLV-CMV::EPG-IRES-hrGFP). mCherry controls were prepared by transfecting neurons with lentivirus construct pLV-CMV::IRES-mCherry. Seven days post transfection, we used (fura-2/AM) calcium imaging to visualize neuronal responses. Post co culture, we wanted to characterize our neuronal co culture using specific neuronal (class-III tubulin, tuj1) and glial cell markers (GFAP). Immunocytochemistry revealed that the coculture show immune reactivity to GFAP and TUJ1 (Fig. 3A,B) in EPG transfected neurons **(**Fig. 3C**)** confirming that the coculture consists of the desired neuron-glia population required for this study. Magnetic field was induced by a custom made electromagnet system that delivered 5 ms pulses at a rate of 10 Hz and induced a field of 50–70 mT. Figure 3E shows a single cortical neuron transfected with pLV-CMV::EPG-IRES-hrGFP showing a GFP signal throughout the cell body and processes. Figure 3F shows labeling of EPG+ neurons with fura 2-AM labeling post 1 hour. Figure 3G demonstrates that magnetic stimulation increased fura-2AM intensity indicating an increase in [Ca^2+^]i. Figure 3H shows raw data traces of fura 2-AM imaging of EPG expressing neurons responses to magnetic stimulation (blue bar). EPG positive neurons that responded to magnetic stimulation (n = 224) were raster plotted across time; 77 neurons out this population (34%) responded within the 10 s of magnetic stimulation **(**Fig. 3I). The average response time was 15 ± 0.5 s. Representative responses of EPG expressing neurons, mCherry expressing neurons (control), non-transfected neurons (control) and EPG expressing neurons in different physiological conditions including 0 Ca^2+^ external solution, 0 Ca^2+^ + EGTA external solution and with the inhibitor thapsigargin, after magnetic stimulation are shown in Fig. 3J. Figure 3K shows mean percentage change in fluorescence before and after magnetic stimulation. Only EPG expressing neurons show increase in fura-2AM fluorescence after magnetic stimulation. Magnetic stimulation induced a 144.8 ± 14.1% mean increase in [Ca^2+^]i in EPG-expressing neurons (n = 224; p < 0.0001). Non transfected control neurons (0.50 ± 0.424%, n = 495) and mCherry expressing neurons (0.14 ± 0.84%, n = 80) did not show any significant change in response to stimulus. fura-2AM imaging of EPG expressing neurons incubated with external 0 Ca^2+^ (2 ± 0.98%, n = 475) and with 0 Ca^2+^ and EGTA (0.84 ± 0.64%, n = 175) did not show significant changes in fluorescence intensity. We then incubated EPG expressing neurons with the SERCA pump blocker, thapsigargin, at a concentration of 1 µM at 37 °C for 1 hour. Subsequent imaging of the neurons did not result in a significant change in fura-2AM fluorescence (0.50 ± 0.3%, n = 308). Together, these results demonstrates that remote activation of EPG in neurons can induce significant increases in [Ca^2+^]i, indicative of neuronal excitability, only in EPG expressing neurons. Similar to the results obtained in HEK293T cells, the neuronal cultures required physiological extracellular and intracellular calcium levels in order to respond to magnetic stimulation. Supplementary Fig. S6 demonstrates that this phenomenon was further confirmed in mouse neural cultures and also by using genetically encoded calcium indicator (GCaMP6s). Furthermore, we wanted to further confirm that the GFP fluorescence of the EPG positive neurons does not interfere with fura-2/AM fluorescence. We found that cells containing GFP (EPG positive) have the same fura-2/AM fluorescence as non-transfected ones (Supplementary Fig. S7). Therefore, the use of the EPG transfected cells expressing GFP as a marker of successful transfection is compatible with the use of fura-2/AM to measure intracellular [Ca^2+^].

### Wireless activation of EPG in a rodent model

**Figure 4:**
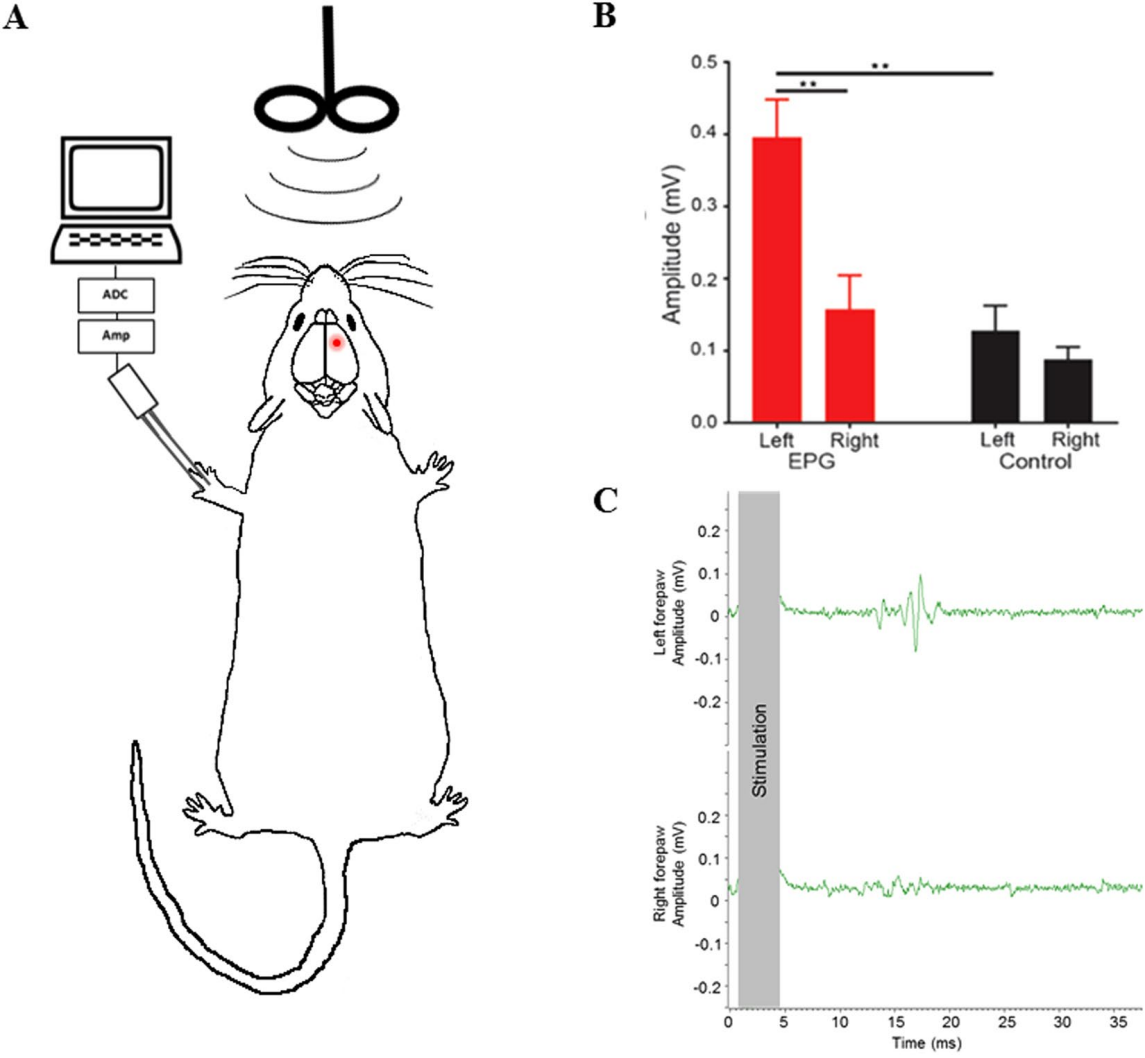
Wireless magnetic stimulation in EPG-expressing rats induces large muscle response. **(A)** A cartoon depicting the *in vivo* setup. **(B)** Magnetic stimulation over the cortex caused a significant increase in the amplitude of the MEP responses in the left forelimb of rats expressing EPG in the right M1. Control rats had minimal MEP responses. (**p < 0.005) **(C)** Representative traces of left (contralateral to EPG expressing M1) and right (ipsilateral to EPG expressing M1) forelimb MEP responses to magnetic stimulation.

Finally, we targeted the rodent limb motor system, which is a well-studied circuit with validated and quantifiable assays for activation. Stereotaxic injection of virus containing EPG (pAAV2-CaMKII::EPG-IRES-hrGFP) was targeted into layer 5 motor neurons in the right primary motor cortex (M1) of the limb area of adult rats (n = 10). Control rats were injected with virus containing only GFP (n = 5). Responses to stimulation of M1 were measured by Motor Evoked Potentials (MEP) of the forelimbs two to three weeks after stereotaxic injections. MEP responses were recorded in both right and left forelimb simultaneously. TMS coil to deliver the magnetic stimulus was positioned over the midline of the rat’s head **(**Fig. 4A**)**. The baseline responses can be observed in the MEP of the contralateral limb in EPG negative rats. Furthermore, the MEP of the limb contralateral to the un-injected limb demonstrate baseline response. In rats that expressed EPG in the right M1, wireless stimulation induced MEP responses in the contralateral, left forelimb (0.39 ± 0.05 mV) that were significantly greater than MEP responses recorded in the ipsilateral, right forelimb (0.15 ± 0.04 mV, p < 0.005, Student’s t-test) (Fig. 4B). In control rats expressing only GFP in the right M1, stimulation induced minimal MEP in the contralateral forelimb which was significantly less compared to the experimental group (0.12 ± 0.03 mV, p < 0.001), and was similar to the MEP response observed in the ipsilateral forelimb (0.08 ± 0.01 mV, p > 0.30) **(**Fig. 4B**)**. The average response latency was 11.3 ± 1 ms, consistent with reports of MEP delays recorded with other brain stimulation methods. These results demonstrate that wireless EPG activation produces larger, measurable behavioral output *in vivo*.

## Discussion

The Earth magnetic field varies between 22 µT to 50 µT and it is the highest at the poles and lowest around the equator. In addition, there are local variations as well as external effects due to magnetic storms on the sun^42,43^. Many animal species can sense small changes in magnetic field^44^, a feature sophisticatedly developed in evolution for navigation and avoidance of predators. However, the mechanisms allowing magnetic sensation are largely unknown.

One such organism known for decades to respond to magnetic fields is the fish *K. bicirrhis*. Here we demonstrated that *K. bicirrhis* senses both static and alternating magnetic fields. We identified EPG as a protein that is responsive to EMF, although the exact mechanism of action and how EPG activity mediates behavior in the *K. bicirrhis* are unknown. Previous measurements of stimulus-evoked changes in intracellular Ca^2+^ using fura-2/AM in the ampullary electroreceptor organ showed that a transduction current depolarizes the apical membrane, and it leads to stimulation of presynaptic Ca^2+^ channels and activation of the synapse^18^. The discovery of EPG suggests a possible signal transduction mechanism that induces depolarization of the electroreceptor organ, and hence, this can facilitate greater understanding of the fish’s physiology and electromagnetic-sensitive capabilities.

It is of great interest to understand the mechanisms by which the EPG senses magnetic signals and translates them into biochemical signals. It is particularly challenging given that there is no precedence of a similar biological system. One approach to investigate the EPG structure and function is through bioinformatics analysis. The analysis revealed the presence of residues in the extracellular domain that may facilitate protein-protein interactions. Therefore, we hypothesize that the EPG protein might have extracellular binding partners. The probable interacting partners of other UPAR_LY6 domain members are (a) UPAR_LY6, (b) Kringle, (c) V-set, (d) Thiol_cytolysin, and (e) Somatomedin_B domains^45^. Since the UPAR_LY6 domain may interact with other UPAR_Ly6 domains, the EPG proteins may form homo-oligomers due to interactions between their extracellular disk-like domains as suggested by Supplementary Fig. S3. Other possible interactions of the UPAR_LY6 domain with Kringle, Somatomedin-B, and Ig-like V-set domains suggest that EPG may also interact with soluble proteins in the extracellular space as well as extracellular matrix proteins. When expressed in neurons, the extracellular UPAR_LY6 domain of EPG may also bind to the Ig-like folds of the extracellular domains (V-set domains) of beta 1 or beta 2 subunits of voltage-gated sodium channels^46–49^. The absence of any known signaling domain associated with EPG leads us to believe that EPG may function mainly as a cell surface protein binding to extracellular partners or extracellular domains of nearby trans-membrane proteins. Our bioinformatics findings show a putative glycosylation site that is in agreement with one hypothesis suggesting that the *K. bicirrhis* sense electromagnetic fields via glycosylated ion channels^50^. Alternatively, the high number of cysteine residues could imply that the EPG is an iron-sulfur protein^51^, even though a conventional iron binding was not identified and our biochemical measurements shows no traces of iron. Recently, a metallic like conductivity was identified in bacteria in proteins rich with aromatic amino acids^52,53^, and finally, it was shown that bacterial enzyme kinetics are affected by low magnetic field via spin-orbit coupling when it binds to Xenon gas^54^. Therefore, it should be noted that a complete explanation of the EPG action has yet to be determined.

In addition to understanding the biological basis of magneto-sensation, there is a great demand for new non-invasive neuromodulation technologies. Recently, efforts to remote control protein production *in vivo* using radio-waves and magnetic field heating of iron nanoparticles have been reported^12,13^. For example, a mammalian temperature-gated calcium channel (TRPV1) coupled to calcium-dependent insulin gene transcription was bioengineered to be activated by radio-waves^11^. Two recent studies reported the engineering of artificial chimeric magneto-sensors where ion channels were fused to ferritin, which is an intracellular protein that stores iron-. Two main theories have been suggested for sensing the Earth magnetic field: The first is based on magnetic nanoparticles and the second is sensing via magneto-chemical reaction, in which different species of radical pairs (different in their spin state) are formed in the presence or absence of magnetic field. A recent study suggests a combination of the two mechanisms in which a complex of two gene producst (*MagR*, that forms magnetic nano-crystals and *Cry*, a cryptochrome photoreceptor) form a “magnetic compass”^55^. However, this model depends on the presence of light for activation. Nevertheless, the above magneto-sensors have raised controversies^56^. Another study implicates the involvement of Kir4.2, BK and CaV1.3 channels in electro-sensing. They report a two-molecule sensing mechanism in which KCNJ15/Kir4.2 couples with polyamines in sensing weak electric fields^57^. In contrast to electromagnetic reception, electrical reception is based on a mechanism of interaction of positively charged intracellular moleculaes with K+ channels^57^. It appears that in both cases there is a K+ channel that is activated in response to minute electrical changes^57,58^. In one case there is a need of binding of polyamines (spermine and spermidine) to slow down the channel activity1 and in the other there’s an insertion of 4 positively charge amino acids (KKKER)2. The mechanisms reported here is novel though it is to be noted that there might be differences in electro- and magnetic field sensing Another gene that is associated with response to magnetic field is the TAX-4 cyclic nucleotide-gated ion channel from the nematode *Caenorhabditis elegans*^16^. The differences between this gene and the EPG suggest that different organisms developed alternative evolutionary strategies for navigation according to electromagnetic fields cues.

Calcium imaging experiments in both HEK293T cells and neurons expressing-EPG showed a delay in the order of seconds in the calcium responses to magnetic stimulation. It is not unlikely that with further tuning of the stimulation parameters, this delay can be shortened. On the other hand, it may be that the EPG technology is not capable of inducing neuronal responses in the order of milliseconds; if the EPG is a membrane associated protein which is connected to an ion channel, and/or work through second messengers to affect neuronal activity, its kinetics could be in the order of seconds. Nevertheless, even with temporal delays in the order of seconds, a non-invasive technology that will allow a transient, cell-specific, and location-specific modulation of neuronal activity would be tremendously powerful.

We successfully expressed EPG in neurons *in vitro*, and we also expressed EPG in the rat’s brain *in vivo*. In the latter, we observed muscular function responses in the forelimb when EPG expressed in the motor cortex was remotely activated. Therefore, neuronal activation by EPG produces a measurable behavioral output. The discovery of EPG as a putative protein responsive to EMF can open new avenues of application of remote controlling cellular activity both in the central nervous system and other non-neuronal systems including the heart, smooth and skeletal muscles, and glial cells. In addition, EPG could be expressed under different promoters enabling cell-specific targeting *in vivo*. Thus, EPG technology could provide an exciting and valuable tool for studying neural activity at the network, cellular, and molecular levels.

## Materials and Methods

All animal procedures were conducted in accordance with the NIH *Guide for the Care and Use of Laboratory Animals* and approved by the Johns Hopkins University Animal Care and Use Committee.

### Fish behavioral experiments

*K. bicirrhis* fish were housed in a 30-gallon tank which was kept at 25 °C. Ammonia, nitrite and pH levels were tested daily. Fish were fed 2–3 times a day with dry flakes. During the experiments the sides of the tank were covered. A behavioral session consisted of 8–10 single trials including control trials with sham stimulus. Successful trials were characterized by the fish swimming away from the EMF. Video recordings of the trials were analyzed using Snagit11 software. Fish location and movement was determined by using the software to mark the end of each fish’s tail prior to the start and during each second of the trial.

### Construction of the cDNA library

Total mRNA was extracted from freshly dissected anal fins of 80 anesthetized glass catfish using the FastTrack 2.0 mRNA Isolation kit (Life Technologies). The cDNA library was constructed in pDONR222 using the CloneMiner II cDNA Library Construction kit (Life Technologies). The final cDNA library was cloned into pcDNA-DEST40 by LR recombination, transformed into One Shot TOP10 Chemically Competent cells (Life Technologies), and stored as glycerol stocks in 500 µL aliquots at −80 °C. The cDNA sub-libraries were constructed by replica plating. A 500 µL glycerol stock of the total cDNA library was added to 5 mL LB (Quality Biological) containing 100 mg/mL Ampicillin (Sigma-Aldrich) and shaken at 225 rpm, 37 °C for 1 hour. The total volume was evenly plated on ten 10-cm Ampicillin plates (Quality Biological) and incubated overnight at 37 °C. The following day, the ten plates were replica plated to a second set of ten plates using nitrocellulose membranes and each nitrocellulose membrane was submerged in a flask containing 50 mL LB. The plates and flasks were incubated overnight at 37 °C, cDNA sub-libraries were purified from the flasks’ inoculums, and glycerol stocks were prepared.

### cRNA Production

Sub-library and individual cRNAs were transcribed using PmeI-digestion and the mMESSAGE mMACHINE T7 ULTRA kit (Life Technologies). After transcription, the poly (A) tailing reaction and DNase I treatment were performed according to the manufacturer’s instructions. The cRNA was purified by either phenol:chloroform extraction followed by isopropanol precipitation or LiCl precipitation, and then dissolved in RNase-free water.

### Oocyte microinjection and two-electrode voltage clamp

Sub-library and individual cRNAs were screened by two-electrode voltage clamp (TEVC). Stage V/VI oocytes harvested from *Xenopus laevis* as reported previously^59^ were injected with 10 to 200 ng of cRNA and maintained at 16 °C in ND97 solution (in mM): 96 NaCl, 2 KCl, 1.8 CaCl_2_·2H_2_O, 1 MgCl_2_·6H_2_O, 5 HEPES, pH 7.5/NaOH). Control oocytes were injected with 50 nL water and incubated in ND97. Three days post-injection, TEVC was performed (Clampex 9.2) by impaling two electrodes (WPI) filled with 3 M KCl with a resistance < 1 MΩ. Recordings were low-pass filtered at 300 Hz. Oocytes were held at −40 mV for 232 ms then voltage-clamped between −100 and 40 mV in 20 mV steps lasting 1.6 ms each and then returned to −40 mV for 230 ms (Oocyte Clamp OC-725A, Warner Instruments). Recordings were made in various bath solutions with and without stimulation. All experiments were conducted at room temperature (20–23 °C). The standard physiological external solution, ND96, contained (in mM) 96 NaCl, 2 KCl, 1.8 CaCl_2_·2H_2_O, 1 mM MgCl_2_·6H_2_O, 5 mM HEPES, 2.5 mM Na-pyruvate, pH 7.5/NaOH. Offline data analysis was performed on custom MATLAB software (MathWorks).

### Bioinformatics

Comprehensive searches for matches for the EPG cDNA with the non-redundant genomic (nr/nt), and the expressed sequence tags (dbESTs) were done using BLASTN and TBLASTX softwares respectively, using the default settings. The cDNA sequence of EPG was then translated in-silico using the EMBOSS translate software tool (standard genetic code), and searched against the non-redundant protein database (nr) using DELTA-BLAST to identify evolutionarily conserved domains. Representative proteins resulting from the DELTA-BLAST and TBLASTX searches were selected to construct a phylogenetic tree of the EPG protein showcasing its evolutionary relationship with other proteins in the same superfamily. The phylogenetic tree of EPG was inferred by Maximum Likelihood method based on the JTT matrix-based model using MEGA6 software. The percentage of trees in which the associated taxa clustered together is shown next to the branches. Initial tree(s) for the heuristic search were obtained by applying the Neighbor-Joining method to a matrix of pairwise distances estimated using a JTT model. The tree is drawn to scale, with branch lengths measured in the number of substitutions per site. The analysis involved 55 amino acid sequences. All positions containing gaps and missing data were eliminated. There were a total of 34 positions in the final dataset. The amino acid sequences of close homologs of EPG (inferred from the phylogenetic tree) were aligned using MUSCLE (2.0) to predict the putative secondary structure of EPG protein. TMHMM, J-Pred, and Signal-Blast analyses were performed on the amino acid sequence to identify the secondary structure of the EPG sequence. The putative membrane orientation of EPG was predicted using Polyphobius software. Finally, the tertiary structure of EPG was predicted by homology based modeling using SWISS-MODEL software and was visualized using DeepView.

### Cloning EPG into mammalian expression vectors

The open reading frame of EPG was cloned into pcDNA3.1D/V5-His-TOPO (Life Technologies) to tag EPG for antibody detection in biochemistry assays (final plasmid: pcDNA3.1-EPG-V5-His). Briefly, primers for directional cloning containing the KOZAK sequence and start codon were used to PCR amplify the EPG gene and clone into the vector, according to product instructions. cRNA was amplified from this plasmid to verify conserved EPG function. To virally express EPG in brain slices, EPG was digested out from pcDNA3.1-EPG-V5-His and ligated into the multi-cloning site of pAAV-IRES-hrGFP (Agilent) using the *BamH*I and *Xho*I sites.

### RT-PCR analysis

Total RNA was extracted from fish tissue using a Quick-RNA Mini Prep kit (Zymo Research). First strand cDNA was synthesized using a iScript cDNA synthesis kit and oligo dT primers (BioRad) according to the manufacturer’s instructions. A reverse transcription-PCR (RT-PCR) assay was designed for the detection of fish EPG mRNA expression. Forward 5′-CGA TCG CGG AGT CTC TTA CC-3′ and reverse 5′-CGG GGT TGC AGT TGT TTG TG-3′ were used as oligonucleotides with the following cycle parameters: (1) 2 min hot start at 96  °C (2) 35 cycles of 1 min at 94  °C, 45 s at 55  °C and 1 min at 72  °C (4) extension at 72  °C for 15 min.

### Adeno-associated virus (AAV) production

High-titer AAVs were produced using the AAV Helper-Free System (Agilent) and concentrated using the AAV Purification Maxi kit (Biomiga). For virus titer (~1 × 10^6^ − 1 × 10^9^ TU/mL), 10-fold serial dilutions of virus were transduced into HEK293T cells plated at 70–80% confluence in 24-well plates and green fluorescent cells were counted 4 days post-infection. Un-concentrated, low-titer virus was produced by following the same transfection procedure as in high-titer virus. 72-hours post-transfection, cells and supernatant were collected and freeze-thawed three times, centrifuged at 4000 rpm at 4 °C for 15 min, filtered in a 0.45 µm filter flask (Millipore), and stored as 1 mL aliquots in −80 °C. In addition, AAV bearing EPG under the excitatory neurons-specific promoter CaMKII (AAV2-CamKIIa-EPG-IRES-hrGFP) was custom made by Vector Biolabs. Infected cells were identifiable by positive GFP expression. Original virus titer of 1.9 × 10^13 was diluted by a factor of 1000 before application to cell culture wells.

### Custom Lentivirus (LV) production

Lentivirus containing the CMV::EPG-IRES-hrGFP and pLV-CMV::IRES-mCherry for control was custom produced from Cyagen Biosciences (Guangzhou) Inc. at a functional titer of 3.57 ± 2 × 10^9^ TU/mL.

### Custom EPG antibody production

A custom anti-EPG polyclonal antibody was produced at Genscript, NY. Analysis of the amino acid sequence of the EPG protein determined that the fragment between amino acid positions 21 and 133 (AA21–AA133) was the immunogenic part of the protein (GenScript’s OptimumAntigen™ Design Program). After removing the N-terminal signal peptide (AA1–AA20), the protein fragment (AA21–AA133) was expressed in a bacterial system. The expressed protein was then purified and used to immunize two New Zealand rabbits using the conventional immunization protocol. Following 3 rounds of immunization, the production bleeds from the two rabbits were separately affinity purified to isolate the polyclonal antibodies. The isolated antibodies were then quality tested by indirect ELISA.

### Immunocytochemistry, co-localization and statistical analysis

HEK293T cells were transfected with pcDNA3.1 EPG using Lipofectamine 2000 Reagent (Life Technologies, Inc.) and were cultured on glass slides coated with poly-L-lysine (PLL; Sigma- Aldrich). Control cells were untransfected. Briefly, cells were washed with PBS 48 h post transfection, fixed in 4% paraformaldehyde for 30 min, washed three times with DPBS and blocked in 5% NGS in PBS for 1 hour. Cell were permeabilized with 0.2% Triton X-100 (Sigma- Aldrich) for 5 minutes, washed again and double stained with 1:100 mouse anti Pan Cadherin (Sigma-Aldrich) and 1:50 costume made rabbit anti EPG antibody overnight at 4 °C. Then the cells were washed again and incubated in 1:500 goat anti mouse Alexa 488 and goat anti rabbit Alexa 568 (Life Technologies, Inc.) for 1 hour. The nucleus was stained with DAPI (25 ng/mL, Sigma-Aldrich) for 10 min. Images were acquired on a Zeiss AxioObserver stage with 780-Quasar confocal module (Carl Zeiss, Oberkochen, Germany) using 63 × /1.4 PlanApo oil objective.

In order to determine if EPG is localized in the cell membrane, we have co-localized EPG expression with membrane immunostaining. Co-localization of confocal images between two channels in HEK293T transfected cells vs. un transfected control cells was measured using Huygens Essential software (SVI Huygens, The Netherlands). We have quantified the number of pixels that had both green (pan cadherin, a membrane stain) and red (anti-EPG). Two different statistical tests, Overlapping and Pearson correlation coefficients were used to obtain quantitative information about the amount of spatial overlap between two channels.

### Calcium imaging in HEK293T cells

HEK293T cells were transfected with pLENTI- CMV::EPG-IRES-hrGFP (4 μg) and Lipofectamine 2000 (Invitrogen) according to standard protocols. Transfected cells were incubated for 48 hours at 37 °C before prior to imaging. Calcium imaging was performed with transfected cells, plated on glass coverslip dishes and incubated overnight in a humidified incubator kept at 37 °C and 10% CO_2_. Cells were washed three times with calcium imaging solution (CIS) (125 mM NaCl, 2 mM MgCl2, 4.5 mM KCl, 10 mM Glucose, 20 mM HEPES & 2 mM CaCl_2_ adjusted to pH 7.4 (no CaCl_2_ was added for the 0 Ca^2+^ buffer ad was substituted with Na ions). These cells were loaded with 1 μM fura-2-AM for 45 min at 37 °C. Cells were then washed three times with CIS and de-esterified for 30 min at 37 °C. Culture dishes were then loaded into customized imaging chambers and imaged. GFP positive cells were randomly selected from an image field. A magnetic stimulus was delivered using static magnets that had a magnetic field of 50 mT for a period of 10 s. Data were quantified as changes in fluorescence over time post magnetic stimulation. Transfected cells were treated with thapsigargin (Sigma T9033) at a concentration of 1 μM and subsequently imaged 1-hour post incubation.

### Immunohystochemistry

Cortical mixed glial cultures were prepared from P0-P2 ICR (CD-1®; Harlan Laboratories) rat pups. Neuron-glia co-cultures were maintained in complete Neurobasal medium. On Day 4 cells were infected with plenti virus containing the CMV::EPG- GFP and un-infected cells served as control.

For neuronal co-culture characterization, we used rabbit polyclonal anti- neuronal class III β-tubulin (Biolegend; 1:500 dilution) as a neuronal marker, mouse monoclonal anti-GFAP (Dako; 1:500 dilution) as a glial marker and chicken anti GFP (abcam; 1:200 dilution). After overnight incubation, the cover slips were washed thoroughly with DPBS and incubated in donkey anti mouse Alexa 405 (abcam), goat anti rabbit Alexa 594, and goat anti chicken Alexa 488 (Thermofisher Scientific), respectively at 1: 500 dilutions for 1 hour. Images were acquired using an M2 AxioImager upright epifluorescence microscope (Carl Zeiss, Oberkochen, Germany) equipped with an Apotome module and 10 × /0.45 NA and 20 × /0.8 NA objectives, and processed using AxioVision, version 4.8 (Carl Zeiss).

### Primary cortical neuron culture and calcium imaging

Cortical mixed glial cultures were prepared from P0-P2 (Harlan Laboratories) rat pups. The cortex was dissected, minced, and incubated with trypsin (2.5 mg/ml; Sigma, St. Louis, MO) and DNase (0.015 mg/ml; Sigma) in 10 mL of DMEM (Invitrogen) with 25 μM glutamate (30 min, 37 °C). Tissue was triturated, resuspended in 10 ml glial maintenance medium made of DMEM with 10% fetal bovine serum (Hyclone, Logan, UT), and cells were filtered twice through 70 μm pore nylon mesh. Cells were plated and medium was changed 24 h after being plated and then every 2–3 days afterwards. Cortical neurons were prepared from E18 (Harlan Laboratories) rat embryos. The cortex was dissected, minced, and incubated with trypsin and DNase in 10 mL of Neurobasal medium supplemented with B27 (Invitrogen) and 200 nM L-glutamine (Invitrogen) (30 min, 37 °C). Tissue was triturated, resuspended in 10 ml of supplemented Neurobasal medium and cells were filtered twice through 70 μm pore nylon mesh. Neurons were placed on top of a confluent mixed glial bed layer at a density of 0.8–1.0 × 10^5 cells per well. Neuron-glia co-cultures were maintained in complete Neurobasal medium. On Day 5 cells were infected with Lentivirus containing the CMV::EPG-IRES-hrGFP or pLV-CMV::IRES-mCherry for control. Other control cells remained non-infected. Cells were incubated at 37 °C for 3 days post-infection. Neuronal-glia cultures were loaded with 1 μM fura-2/AM (Molecular Probes/Invitrogen; 45 min, 37 °C) in Tyrodes solution (119 mM NaCl, 5 mM KCl, 25 mM HEPES buffer, 2 mM CaCl2, 2 mM MgCl2, 6 g/liter glucose, Adjust pH to 7.4 with NaOH) and then incubated for an additional 30 min at 37 °C to allow for complete hydrolysis of the acetoxy-methyl ester group. An inverted Olympus 1 × 71 microscope with a dual condenser illumination column was used to image fluorescent changes that are proportional to intracellular calcium concentration during stimulation. The percentage change in fura-2 ratio at 340/380-nm excitation relative to values recorded at t = 0 for each neuron was calculated. A magnetic stimulus was delivered using an electromagnet that had a magnetic field of 50 mT for a period of 10 s. Data were quantified as changes in fluorescence over time post magnetic stimulation. Transfected cells were treated with thapsigargin (Sigma T9033) at a concentration of 1 μM and subsequently imaged 1-hour post incubation. For calcium free experiments 0 Ca^2+^ tyrodes was used. For experiments involving additional external Ca^2+^ buffering, 3 Mm EGTA was used.

### EPG-GCaMP6 Imaging

Neuron-glia co-cultures from mice were maintained in complete Neurobasal medium. On Day 5 cells were infected with AAV2 virus containing the CaMKII::EPG-GFP or only CaMKII::GFP for control. Other control cells remained un-infected. Cells were incubated at 37 °C for 3 days post-infection. Neurona-glia cultures were loaded with 1 μM fura-2/AM (MolecularProbes/Invitrogen; 45 min, 37 °C) in Hank’s balanced salt solution with 10 mM HEPES buffer (pH 7.2), and then incubated for an additional 30 min at 37 °C to allow for complete hydrolysis of the acetoxy-methyl ester group. Changes in [Ca^2+^]i were also detected via use of genetically encoded calcium sensors, AAV5-Syn::GCaMP6s-WPRE-SV40 (Penn vector lab, Philadelphia, PA). After neuron-glia cultures had been allowed to mature for 5 days, GCaMP 6s viral vector was administered to a final concentration 1 × 10^6 viral particles/mL. Neurons were imaged eight-days post-transfection. An inverted Olympus 1 × 71 microscope with a dual condenser illumination column was used to image fluorescent changes that are proportional to intracellular calcium concentration during stimulation. The percentage change in fura-2 ratio at 340/380-nm excitation relative to values recorded at t = 0 for each neuron was calculated. GFP-positive neurons that the stimulation induced increases in the fura-2 averaged ratios by at least one standard deviation were considered responsive. Data analysis was performed on custom MATLAB software.

### Stereotaxic AAV injection in the rat brain

Adult Sprague-Dawley rats received stereotaxic injection of AAV2 virus encoding for EPG under CaMKII promoter (pAAV2-CaMKII::EPG-IRES-hrGFP) (n = 10) or AAV2 encoding only to GFP (pAAV2-CaMKII::IRES-hrGFP) (control, n = 5). Surgeries were performed under isoflurane anesthesia. Microinjection needle was placed at the right M1 to target forelimb (AP: + 1.6 mm, ML: +3.0 mm, DV: −1.7 mm) and 3 µL of AAV2 was injected. In addition, P1 Sprague-Dawley rats were stereotaxically injected with 1 µl of AAV virus encoding for EPG or GFP in the right lateral ventricle (*n* = 10; anterior-posterior, 0.8 mm, medial-lateral, 2 mm, dorsal-ventral, 1.8 mm) according to Li *et al*.^7^. Rats were fixed into the stereotaxic apparatus under cryoanesthesia.

### Immunohistochemistry of brain slices

Four weeks after stereotactic viral injection into the lateral ventricles at age P1, P30 injected rats and age matched un-injected control group were perfused with 4% paraformaldehyde (ice cold) and 10% formalin (room temperature) solution respectively. Free floating 50 µm sections were incubated with the anti-EPG polyclonal antibody for 48 hours at 4 °C. The sections were then washed and incubated with a biotinylated secondary antibody (Vector Laboratories, CA) for 1 hour at room temperature. The sections were then processed for Nickel-3,3′-diaminobenzidine (Ni-DAB) staining using Vectastain Elite (Vector Laboratories)^60^. The sections were imaged in an AxioImager M2 (Carl Zeiss AG) using Axiovision Rel 4.8 software for image acquisition.

### *In vivo* motor evoked potential measurements

Adult rats expressing EPG targeted at L5 neurons in motor cortex were initially anesthetized with isoflurane and then anesthesia was maintained on dexmedetomidine (0.1 mg/kg/hr s.c.). The rats were implanted with electromyography (EMG) electrodes in the bilateral biceps (Natus, Pleasanton, CA). A TMS coil was placed over the head with the center of the coil overlying the midpoint of the bilateral motor cortices. Approximately 75% of stimulator output was required to induce resting motor threshold of rat bicep muscle. In order to maximize the detection of TMS induced MEP, maximum output of the machine at 100% was used (approximately 30% of resting motor threshold) throughout the experiment. Electromyography recording was performed with the following specifications: high pass filter at 5 kHz, low pass filter at 500 Hz, sampling rate at 25 kHz. Amplification was performed at 2 × 10^3^ (FHC, Bowdoin, ME) and data was collected using Micro1401-3 (CED, Cambridge, UK) data acquisition unit. Using Spike 2 software (CED, Cambridge, UK), the amplitudes and latency of motor evoked potentials were measured. Amplitude was determined as the height from negative peak to highest peak, and latency was defined as the time point where initial peak occurred following stimulus. Averages from ten sweeps were calculated for each animal.

### Magnetic Stimulation

The field strength of the magnet was measured consistently prior to experiments requiring stimulation. For this purpose, we employed a Gauss meter. Before start of experiments involving fish stimulation, we used a ruler to measure the distance between the fish and the magnetic stimulation source and measured the field strength repeatedly using a gauss meter. We report that our values were consistent. For experiments involving stimulation with HEK cells and cortical neurons, magnetic field was induced by a custom-made electromagnet system that delivered 5 ms pulses at a rate of 10 Hz and induced a field of 50–70 Milli Tesla. The electromagnet was held and positioned using a manipulator system. The cells in the dish was first placed on the microscope and then the field strength of the electromagnet was measured using a gaussmeter from a distance of 1 cm. The Electromagnetic coil is remote controlled by an electrical input. We switch off the input and slowly position the electromagnetic coil above the field of cells. We determined that the distance between the electromagnet and the cells were 1 cm away. We then turn on the stimulus for a period of 10 seconds.

### Statistics

Results and figures show the average ± standard error of mean (SEM). Two-tailed Student’s *t* test and ANOVA was used when appropriate (unless stated otherwise).

### Data availability

The datasets generated during and/or analyzed during the current study are available from the corresponding author on reasonable request.

## Electronic supplementary material

Movie S1 A

Movie S1 B

Supplementary Information
