# Supplementary material for: Wireless control of cellular function by activation of a novel protein responsive to electromagnetic fields
Source: Sci Rep. 2018 Jun 8;8:8764. doi: 10.1038/s41598-018-27087-9 (PMC5993716; doi:10.1038/s41598-018-27087-9)
Supplement: Supplementary file 3 — Supplementary Information [file 41598_2018_27087_MOESM3_ESM.pdf]

Supplementary Information

Wireless control of cellular function by activation of a novel protein responsive to electromagnetic fields

Vijai Krishnan [1,2,8,9#], Sarah A. Park [1,2#], Samuel S. Shin [1,2], Lina Alon [1,2], Caitlin M. Tressler [2,3], William Stokes [1], Jineta Banerjee [1,2], Mary E. Sorrell [1,2], Yuemin Tian [4], Gene Y. Fridman[5], Pablo Celnik[6], Jonathan Pevsner [7], William B. Guggino [4], Assaf A. Gilad [1-3.8,9,10*], Galit Pelled [1,2,8,9,10*]

Movie S1.

a. The movie shows that the *K. bicirrhis* swim away from the source of the EMF. The TMS coil was placed on the right side of the fish tank and induced pulses in a rate of 50 Hz for 10 s.
b. In order to verify that the behavioral response was specific to the EMF stimulus, in control trials the TMS was placed at the same location, but only an audio-recording of the sound has been delivered for 10 s. During these trials fish were indifferent to the sound.

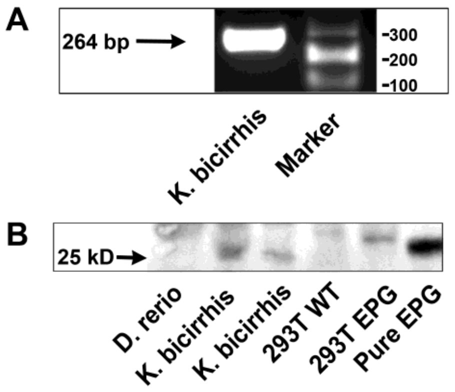

Supplementary Figure S1. EPG expression: (A) RT-PCR analysis was performed using specific primers for fish EPG gene on total RNA isolation from the *K. bicirrhis*. Arrow indicates a 264 base pairs size band corresponding to the fish EPG mRNA. **(B)** Western blot analysis of tissue extract from *D. rerio* (Zebrafish), *K. bicirrhis*, Wildtype HEK 293T (293T WT), HEK 293T expressing EPG (293T EPG) and purified EPG. Molecular weight standards (Biorad) indicate all EPG bands around 25 kD which indicates EPG is expressed as a dimer in all three systems. The molecular weight of the EPG is slightly different due to tags present on the proteins. The protein expressed in 293T cells contains both a hexa-His tag and a V5 tag. The protein expressed in bacterial systems contains a hexa-His tag. **For full length figure of gel and western blot see Supplementary Figure S8**

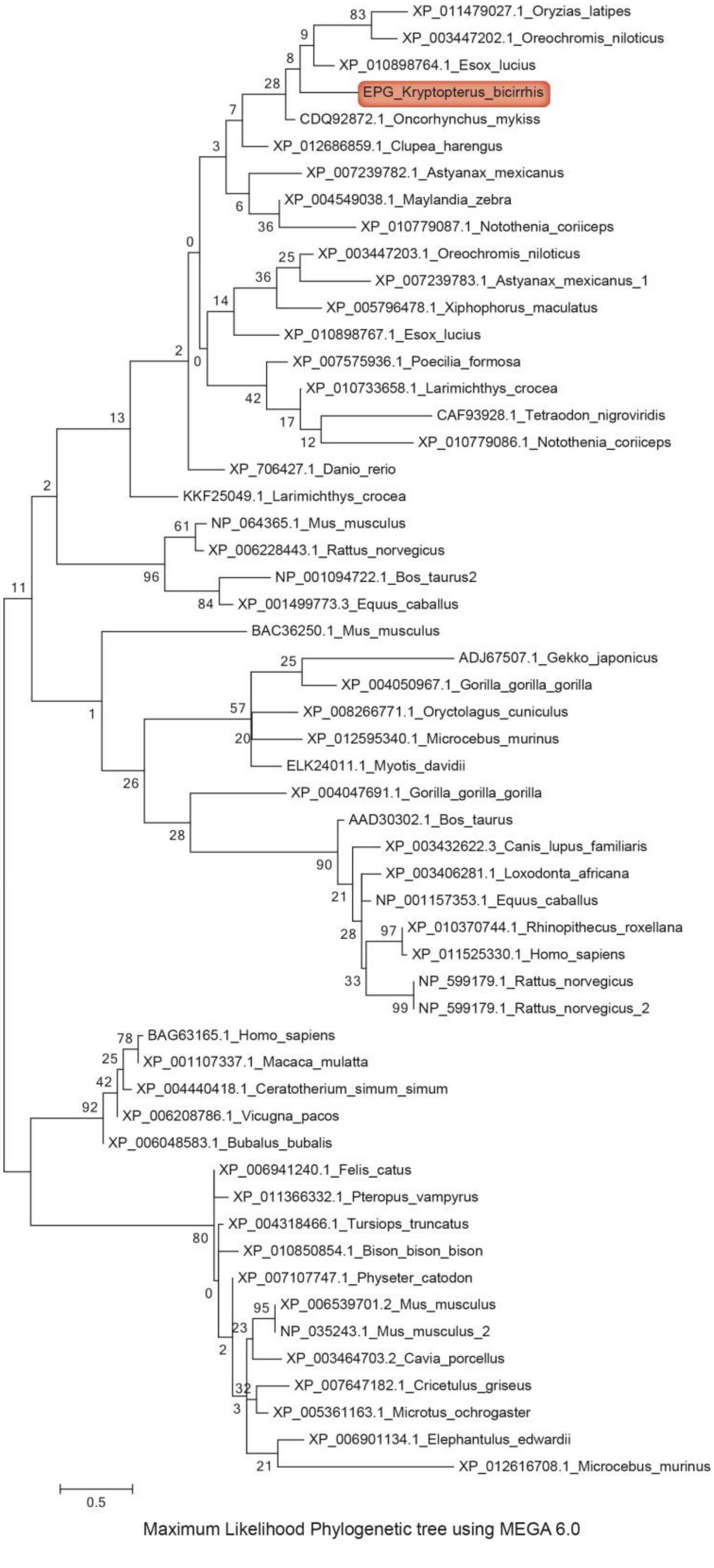

Supplementary Figure S2. Molecular Phylogenetic analysis by Maximum Likelihood method. The phylogenetic relationship of EPG (highlighted in red) inferred using the Maximum Likelihood method

based on the JTT matrix-based model. The analysis compared EPG amino acid sequence with 55 protein sequences from various taxa. The tree with the maximum log likelihood (-2523.94) is shown. The numbers next to the branches indicate the percentage of trees in which the associated taxa clustered together. The tree is drawn to scale, with branch lengths measured in the number of substitutions per site. All positions containing gaps and missing data were eliminated. Evolutionary analyses were conducted using MEGA6 software.

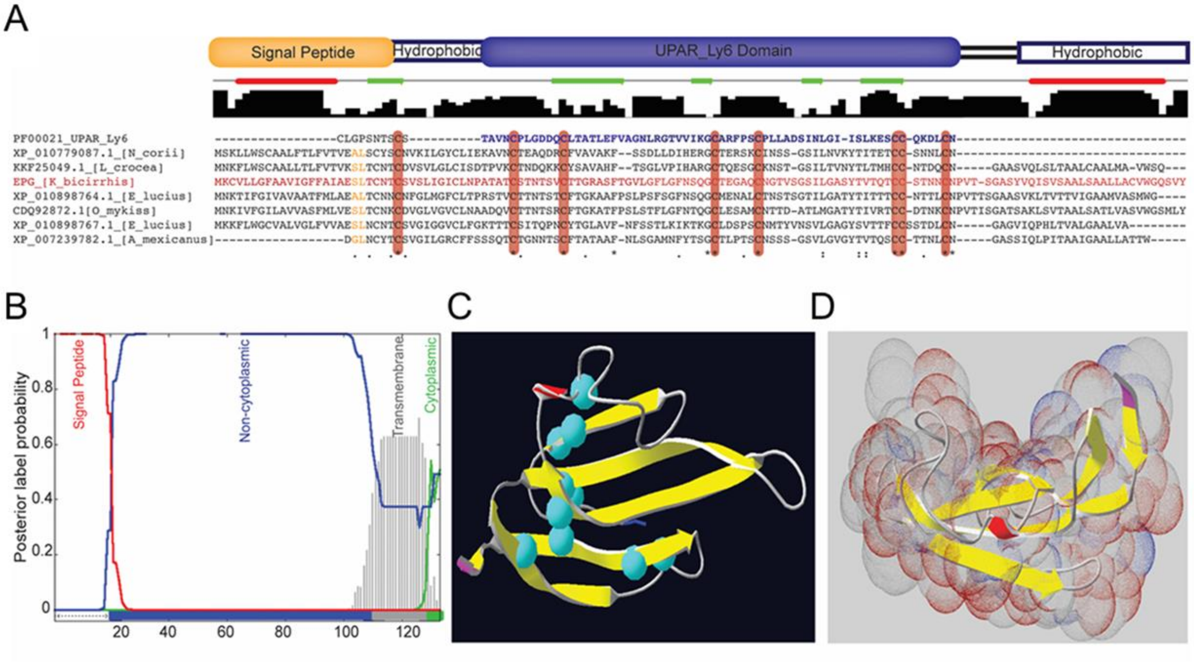

Supplementary Figure S3. Predicting the putative structure and function of the EPG protein. (**A**) The top panel shows a diagrammatic representation of putative domains of EPG based on the PFAM definition of the UPAR_LY6 domain and secondary structure analyses. The middle panel shows secondary structure predictions from JPred software. The bottom panel shows multiple sequence alignment of EPG and six of its closest homologs (as determined by phylogenetic analyses) using MUSCLE (3.8). The consensus sequence of the UPAR_Ly6 (Pfam database accession *PF00021*) is included (residues identified from BLAST searches are in bold and shaded blue). (**B**) Putative membrane orientation of the EPG protein based on PolyPhobius analysis of the primary amino acid sequence. (**C**) Predicted tertiary structure of the UPAR_Ly6 domain in EPG (spanning residues 17-104) using homology prediction from SWISS-MODEL software. The panel shows a ribbon and sheet model highlighting the positions of cysteine residues (cyan) and beta sheets (yellow arrows showing antiparallel orientation). The N terminus is at the bottom left and the C terminus is at the top left. The closest known structures identified by SWISS-MODEL included known structures of monomers of urokinase plasminogen activator surface receptor (eg. SWISS-MODEL template library (SMTL) ID: 1ywh.1, SMTL ID: 1ywh.1.G, and SMTL ID: 3laq.2.B). (**D**) Accessibility plot of EPG generated using DeepView software highlights the concave nature of the UPAR/Ly-6 domain (rotated view from the C terminal end looking down the groove).

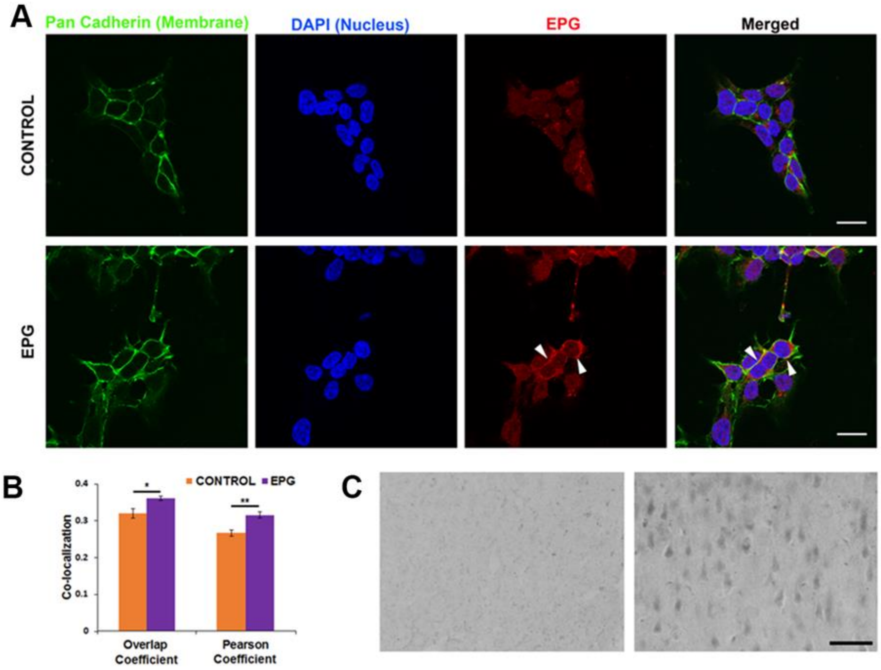

Supplementary Figure S4. Immunostaining characterization of EPG expression in mammalian cells and in the rodent brain. (A-C) HEK293T cells transfected with pcDNA3.1-EPG (A-B; lower panel; EPG) and control un-transfected cells (A-B; upper panel; CONTROL) were stained with polyclonal anti-EPG antibody that recognizes specific epitopes of the EPG (red), anti-Pan Cadherin, a plasma membrane marker (green), and DAPI for nuclear DNA staining (blue). (A) Confocal images demonstrate expression of EPG in the cells periphery (arrowheads) (B) Quantifying the degree of co-localization between red and green channels of control and EPG transfected cells, using both the Mander's overlap and the Pearson correlation coefficients (Mean ± SEM). (C) Stereotaxic injections of pAAV2-CaMKII::EPG-IRES-hrGFP were performed in the rat brain. Immunostaining images in the primary somatosensory cortex showing cortical EPG expression in fixed brain sections using polyclonal anti-EPG antibody (right), and non-injected brain (left; Scale bar = 50 μm).

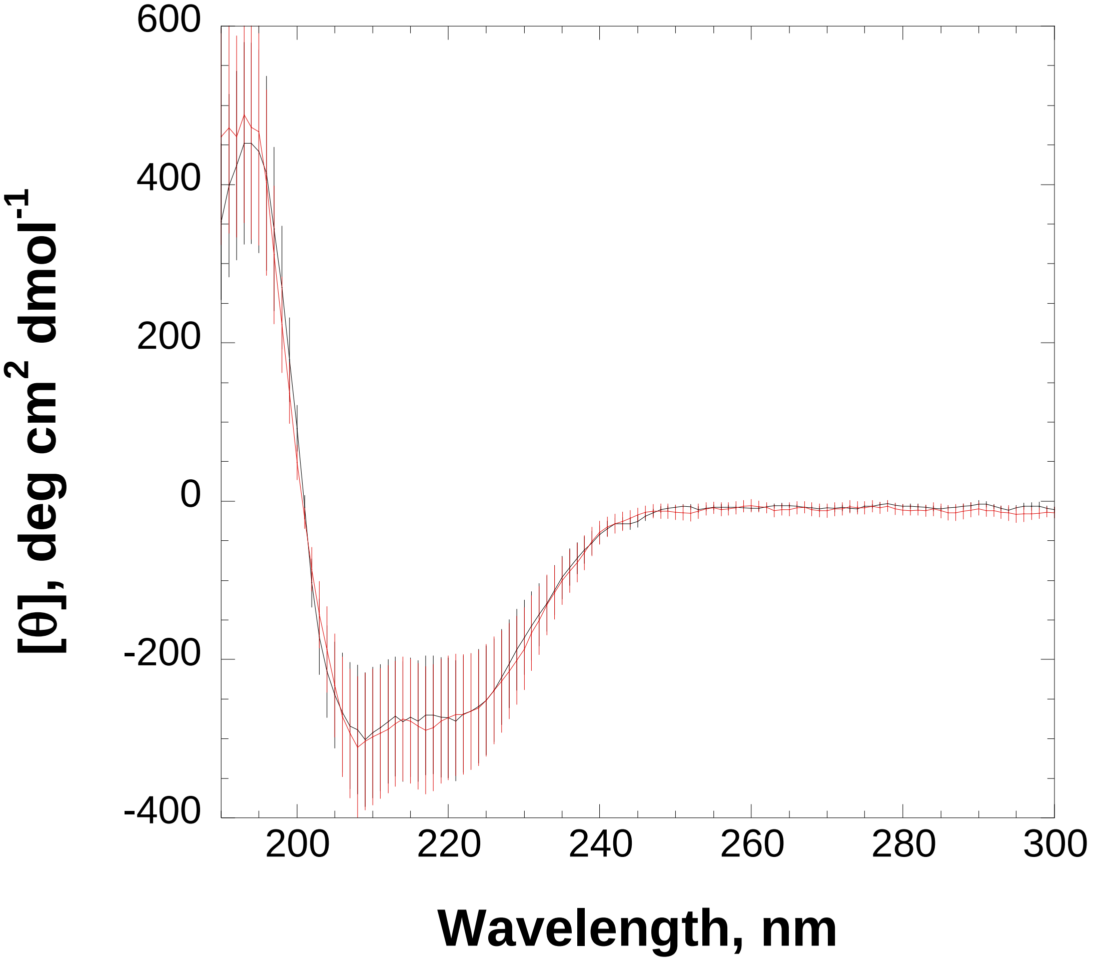

Supplementary Figure S5: CD Spectra of EPG: CD spectrum of purified EPG protein in 20 mM sodium phosphate (pH 7.4) with 100 mM NaF at room temperature. Data was collected in the absence of a magnetic field (black) and in a 25 mT magnetic field (red). Data is representative of at least three independent trials and error bars indicate standard error.

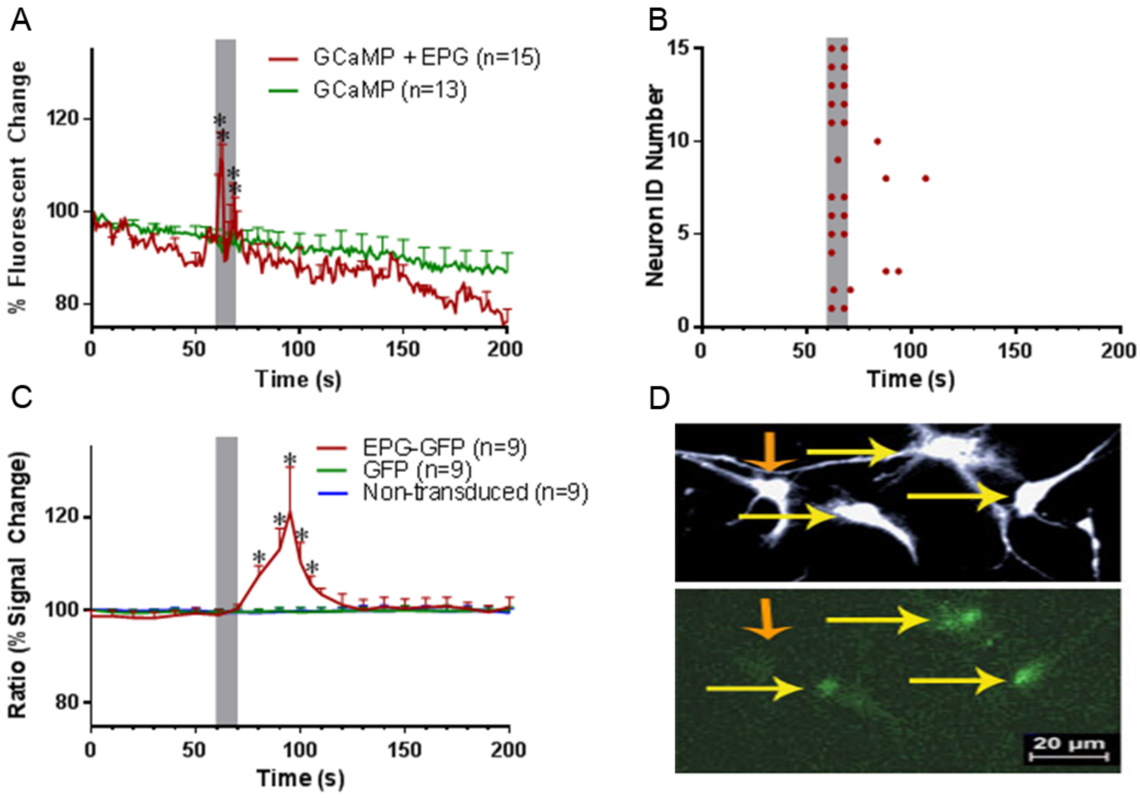

Supplementary Figure S6: Magnetic stimulation of EPG in neurons induces significant increases in $[Ca^{2+}]i$. (a) Primary cortical mixed neuron and glia cultures were transduced with viral constructs for EPG tagged with mCherry under the CamKII promoter, and GCaMP6s expression. Static magnetic field was applied for 10 s (gray bar). Significant increases in $[Ca^{2+}]i$ compared to baseline values were measured only in neurons expressing EPG-GFP (* $p<0.0005$, Student's t test). (b) Calcium spikes raster plot of the EPG and GCaMP6 expressing neurons. (c) Primary cortical mixed neuron and glia cultures were transduced with either AAV expressing the EPG tagged with GFP or AAV expressing GFP only, under the CaMKII promoter. Non-transduced cells were also used as a control. Cells were loaded with fura-2 calcium indicator dye before the experiment. A 20 Hz alternating magnetic field was applied for 10 s (gray bar). Significant increases in $[Ca^{2+}]i$ compared to baseline values were measured only in neurons expressing EPG-GFP (* $p<0.0005$, Student's t test).(d) Examples of neurons loaded with fura-2 that were GFP-positive i.e., expressing EPG, are indicated by the yellow arrows, and an example of a GFP-negative neuron is indicated by the orange arrow.

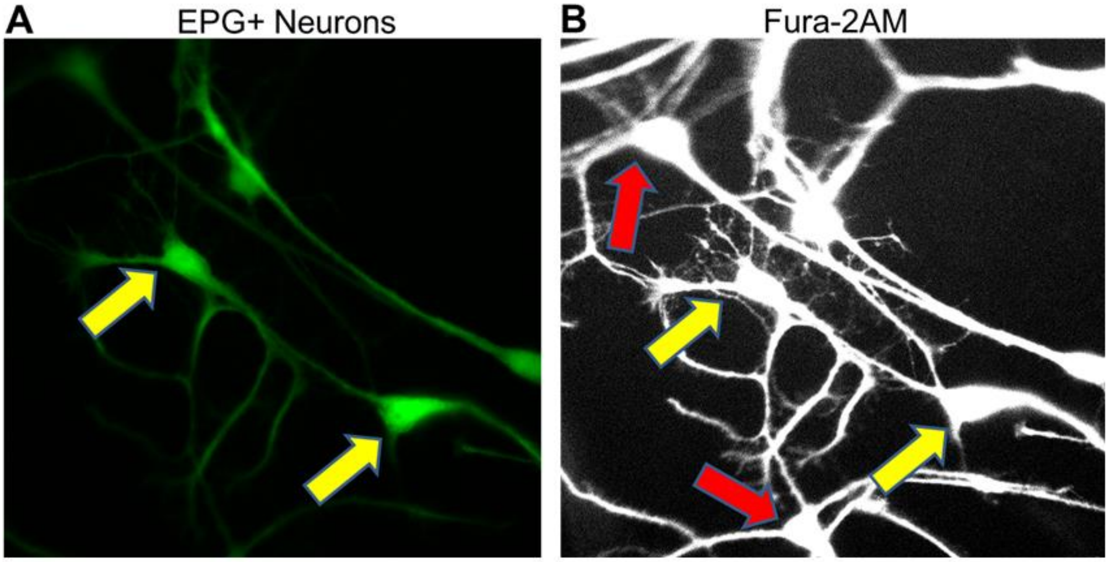

Supplementary Figure S7: Rat cortical neurons transfected with the (pLV-CMV::EPG-IRES-hrGFP) vector. (A) GFP fluorescence showing EPG positive transfected neurons (yellow arrows). (B) Fura-2 fluorescence, with the same field of view with both transfected (yellow arrows) and non transfected cells (red arrows) showing similar levels of fura-2 fluorescence. (arrows show the same cells as indicated in A). X40

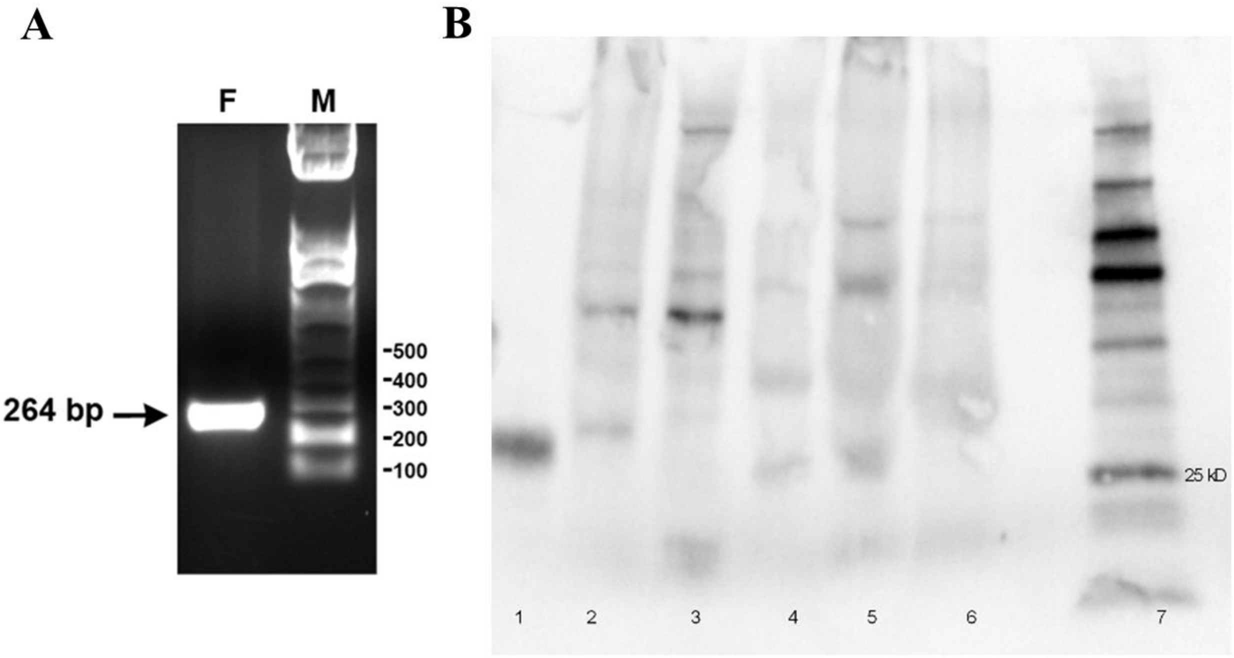

Supplementary Figure S8: EPG expression: (a) RT-PCR analysis was performed using specific primers for fish EPG gene on total RNA isolation from the Kryptopterus bicirrhis (glass catfish). Arrow indicates a 264 base pairs size band corresponding to the fish EPG mRNA. M=marker. (b) Western blot analysis of purified EPG (Lane 1), HEK 293T expression of EPG (Lane 2), Wildtype HEK293T (Lane 3), Glass catfish (Lane 4 and 5), and zebrafish (Lane 6). Molecular weight standards (Biorad) indicate all EPG bands around 25 kD which indicates EPG is expressed as a dimer in all three systems.
